# Changes in College Students Mental Health and Lifestyle During the COVID-19 Pandemic: A Systematic Review of Longitudinal Studies

**DOI:** 10.1007/s40894-022-00192-7

**Published:** 2022-08-03

**Authors:** Chiara Buizza, Luciano Bazzoli, Alberto Ghilardi

**Affiliations:** Department of Clinical and Experimental Sciences, University of Brescia, Viale Europa 11, 25123 Brescia, Italy

**Keywords:** College students, COVID-19 pandemic, Lifestyle, Mental health, Well-being

## Abstract

College students have poorer mental health than their peers. Their poorer health conditions seem to be caused by the greater number of stressors to which they are exposed, which can increase the risk of the onset of mental disorders. The pandemic has been an additional stressor that may have further compromised the mental health of college students and changed their lifestyles with important consequences for their well-being. Although research has recognized the impact of COVID-19 on college students, only longitudinal studies can improve knowledge on this topic. This review summarizes the data from 17 longitudinal studies examining changes in mental health and lifestyle among college students during the COVID-19 pandemic, in order to improve understanding of the effects of the outbreak on this population. Following PRISMA statements, the following databases were searched PubMed, EBSCO, SCOPUS and Web of Science. The overall sample included 20,108 students. The results show an increase in anxiety, mood disorders, alcohol use, sedentary behavior, and Internet use and a decrease in physical activity. Female students and sexual and gender minority youth reported poorer mental health conditions. Further research is needed to clarify the impact of the COVID-19 pandemic on vulnerable subgroups of college students.

**Supplementary Information:**

The online version contains supplementary material available at 10.1007/s40894-022-00192-7.

## Introduction

College students’ mental health has been an increasing concern. The COVID-19 pandemic has brought this vulnerable population into renewed focus. The pandemic has been an important stressor that may have compromised the mental health of college students and changed their lifestyles with significant consequences on their well-being and academic performance. Several studies have shown that college students present poorer mental health compared to their peers in the general population (Kang et al., 2021; Lovell et al., 2015), and the COVID-19 pandemic may have been a stressor that further worsened the mental health conditions of college students. However, the empirical link between COVID-19 pandemic and college students’ mental health has not been established clearly. Although many studies assess the effects of the pandemic on college students, no systematic reviews analyze the changes in their well-being and lifestyles over time. This study addresses this research gap by systematically reviewing the longitudinal studies that investigate the differences in college students’ mental health and lifestyles before and during the COVID-19 pandemic.

The worst mental health condition of college students, compared to their peers, seems to be related to the fact that college students are exposed to a high number of stressors. The academic career is a critical period of life that involves facing new and complex developmental challenges (Ruby et al., 2009). College students are in an uncomfortable position between family expectations, personal achievements, study, and work, and all these factors may contribute to the development or intensification of certain psychological illnesses (Hyun et al., 2007; Sharp & Theiler, 2018). During college years, students are also actively involved in a process of identity formation, influenced by contact with their peers (Adams et al., 2006; Luyckx et al., 2006), with important consequences on their self-esteem and psychological well-being (Cameron, 1999). Isolation and loneliness, as experienced during the COVID-19 pandemic, can be a significant risk factor for good psychological development and for the mental health of adolescents and young adults (Acquah et al., 2016; Adam et al., 2011; Bozoglan et al., 2013; Chang et al., 2014; Christ et al., 2017; Muyan & Chang, 2015; Peltzer & Pengpid, 2017; Shen & Wang, 2019; Zawadzki et al., 2013).

Stressful life events also seem to be a factor that can contribute to the development of mental disorders (Cohen et al., 2019; Meyer-Lindenberg & Tost, 2012; Slavich, 2016). According to psychiatric epidemiological research, most high-prevalence mental disorders emerge during adolescence and early adulthood (de Girolamo et al., 2012; Kessler et al., 2007). As is shown by a recent systematic review of studies assessing differences in mental health among the general population before and during the COVID-19 pandemic, mental health problems increased during lockdown, although without a specific trend (Richter et al., 2021). Some studies have revealed a possible increase in mental health problems among college students during the pandemic (Deng et al., 2021), while other studies have found a decrease in psychological symptoms (Horita et al., 2021; Li et al., 2020b; Rettew et al., 2021). Another important limitation of research on this topic has been its reliance on cross-sectional designs, which does not allow for displaying the evolution of mental health and lifestyle of students over time. Understanding the effects of the pandemic requires an overview of longitudinal studies to highlight changes in psychological symptoms, lifestyle, and well-being during the COVID-19 pandemic in order to compare it to a baseline assessment before the restrictions were imposed.

## Current Study

The COVID-19 pandemic has been a stressor that may have compromised the mental health of college students and changed their lifestyles with important consequences on their well-being. Although research has recognized the impact of COVID-19 on college students, longitudinal studies can contribute greater knowledge on this topic. This systematic review summarizes available data from longitudinal studies examining changes in mental health and lifestyle among college students during the COVID-19 pandemic.

## Methods

### Protocol and Registration

This systematic review was conducted according to the Preferred Reporting items for Systematic Reviews and Meta-Analyses (PRISMA) statement (Moher et al., 2015). The protocol was registered with the Open Science Foundation (OSF) database. The protocol, which includes the research questions, detailed methods, and planned analyses for the review, can be accessed at the following URL: https://osf.io/m6hyg/, 10.17605/OSF.IO/M6HYG (Date of registration: 31 May 2021).

### Inclusion Criteria

This systematic review included longitudinal studies examining changes in mental health and lifestyle among college students during the COVID-19 pandemic. The term “change” is defined as any before-after difference reported in the mental health and lifestyle related variables investigated by the included studies. Data collected before January 2020 (for Wuhan and Hubei province, China), February 2020 (for the rest of China), and March 2020 (for the rest of the world) are considered “before” the COVID-19 pandemic. Data collected after those dates are considered “during” the COVID-19 pandemic.

Articles were included if they satisfied the following criteria: (a) written in English; (b) published in peer-reviewed journals; (c) reported original data; (d) assessed college students’ mental health and lifestyle before and during the COVID-19 pandemic; (e) focused only on college students (age ≥ 18 years); (f) had a follow-up drop-out rate < 60%. This review excluded case reports, dissertations, protocols, reviews, case series studies, unpublished studies, and studies in languages other than English.

### Information Sources and Search Strategy

A systematic search was performed in PubMed, EBSCO (including PsycINFO, PsycARTICLES, Psychology and Behavioral Sciences Collection), SCOPUS and Web of Science, using the following keywords: (“university student*” OR “college student*” OR “undergraduate student*”) AND (COVID-19 OR coronavirus OR SARS-CoV-2) AND (longitudinal OR before OR after OR effect* OR impact) AND (well-being OR “mental health”). Search strategies differed depending on the bibliographic database; the search strategies are shown in Supplementary Table 1. The literature search included all articles that were published up to May 2021.

### Study Selection

Two authors (C.B. and L.B.) screened independently the article titles and abstracts for inclusion and exclusion criteria and extracted data from all full-text articles selected. Any disagreements in data extraction process were negotiated between the two authors.

### Data Extraction

Data were collected in a specific data extraction form, reporting the following items: authors and country of the study; sample characteristics (e.g., sex, average age, course of study, academic year, and ethnicity); timing of assessments; drop-out rate; assessment tools; outcome, and main study results. The two authors abstracted data independently, and any disagreement was revolved by consensus. Because the studies differed in methodology, a narrative synthesis was planned.

### Methodological Quality Assessment and Risk of Bias

The quality assessment of studies was performed independently by two authors (C.B. and L.B.) and confirmed by a third author (A.G.) by using an adapted form of the Newcastle–Ottawa Scale (NOS) for cohort studies (Wells et al., 2011). The NOS is one of the best-known scales for assessing quality and risk of bias in observational studies. The NOS included six items, categorized into three dimensions, including selection, comparability, and outcome. For each item, a series of response options is provided. A star system is used for the semi-quantitative assessment of study quality, such that the highest quality studies are awarded a maximum of one star for each item. The NOS ranges between zero and six stars: two stars or fewer indicating a low quality study, three stars indicating a medium quality study, and four or more stars indicating a high quality study. The selection dimension evaluates the representativeness (item 1) and the sample size (item 2); comparability evaluates whether the confounding factors are controlled (item 3); outcome evaluates the assessment of the outcomes (item 4), whether the statistical analysis is clearly described and appropriate (item 5) and whether the drop-out rate is reported (item 6).

## Results

**Fig. 1:**
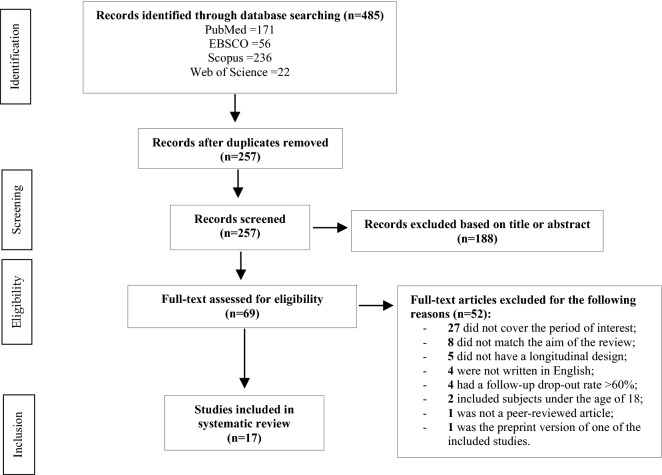
Prisma flowchart showing study selection stages

As shown in Fig. 1, out of 485 articles generated by the preliminary search strategy, 228 were duplicates, and 188 were excluded based on title and abstract, as they were irrelevant to the study criteria. After reading the full text, a further 52 articles were excluded: of these, 27 did not cover the period of interest, eight did not match the aim of the present review, five did not have a longitudinal design, four were not written in English, four had a follow-up drop-out rate > 60%, two included subjects under the age of 18, one was not a peer-reviewed article and one was the preprint version of one of the included studies. In the end, 17 studies were included in the systematic review.

### Methodological Quality and Risk of Bias Within Studies

The quality assessment based on the NOS tool is reported in Table 1. The quality of the studies was quite low, with a consequent high risk of bias; specifically, ten of the included studies were judged low quality, three were judged medium quality, and four high quality.

**Table 1:** NOS Scale

Study	Selection		Comparability	Outcome			Total	Quality
	Item 1	Item 2	Item 3	Item 4	Item 5	Item 6		
Bussone et al. (2020)				*	*	*	3	Medium
Charles et al. (2021)				*	*		2	Low
Copeland et al. (2021)				*	*		2	Low
Dun et al. (2021)	*		*	*	*	*	5	High
Elmer et al. (2020)				*	*		2	Low
Fruehworth et al. (2021)				*	*		2	Low
Hamza et al. (2021)				*	*		2	Low
Horita et al. (2021)				*	*		2	Low
Huckins et al. (2020)				*	*	*	3	Medium
Li et al. (2020a)				*	*	*	3	Medium
Li et al. (2020b)		*		*	*	*	4	High
Meda et al. (2021)				*	*		2	Low
Rettew et al. (2021)				*	*		2	Low
Saraswathi et al. (2020)			*	*	*	*	4	High
Van Zyl et al. (2021)				*	*		2	Low
Wilson et al. (2021)				*	*		2	Low
Zhang et al. (2020)			*	*	*	*	4	High

The NOS includes 6 items: representativeness (item 1); sample size (item 2); comparability (item 3); assessment of the outcomes (item 4); appropriateness of statistical analysis (item 5); drop-out rate assessment (item 6)

### Studies and Sample Characteristics

Table 2 shows the characteristics of the included studies. All studies were longitudinal cohort studies. Seven studies were conducted in the USA (Charles et al., 2021; Copeland et al., 2021; Fruehwirth et al., 2021; Huckins et al., 2020; Rettew et al., 2021; Wilson et al., 2021; Zhang et al., 2020), three in China (Dun et al., 2021; Li et al., 2020a, 2020b), two in Italy (Bussone et al., 2020; Meda et al., 2021), one in the Netherlands (Van Zyl et al., 2021), one in Switzerland (Elmer et al., 2020), one in India (Saraswathi et al., 2020), one in Canada (Hamza et al., 2021), and one in Japan (Horita et al., 2021).

**Table 2:** Description of reviewed studies

Author(s) and Country	Sample N (% female) MA (SD) Ethnicity	Course of study Academic year	Timing of assessments	Drop-out rates at follow-up	Assessment tools	Outcome(s)
Bussone et al. (2020) *Italy*	Same sample 68 (80%) NA NA	NA NA	T1 = 6 months (on average) before the COVID-19 pandemic T2 = 23/04/2020–04/05/2020	None	SCL-90-R PSS-10 STAI-Y RQ PBI	Significant increase in phobic anxiety, depression, psychological distress and perceived stress during lockdown. Parental bonding and attachment style modulate the psychological status during lockdown
Charles et al. (2021) *USA*	Different samples 254 168 352 (84%)^a^ 21.1(NA) White 61.2%	Psychology NA	T1 = 09/2019–11/2019 T2 = 04/2020–05/2020 T3 = 10/2020–11/2020	NA	Questions about COVID-19 CCSM PSS-10 AUDIT	Students in spring 2020 reported more mood disorder symptoms, perceived stress and alcohol use than did pre-pandemic participants, and worry about COVID-19 was negatively associated with well-being. By fall, 2020 symptoms had largely returned to pre-pandemic levels. White students reported a greater effect of the pandemic on well-being than African and American students
Copeland et al. (2021) *USA*	Same sample 576 (75.5%) NA White 90.8%	NA	SS = beginning of spring semester 2020 SE = end of spring semester 2020	14.6%	COVID-19 survey; BPM/18–59; EMA (5 well-being items, 2 mood items)	Problems in externalization and attention increased after the start of COVID-19. Nightly surveys of both mood and daily well-being behaviors were negatively affected by COVID-19 pandemic
Dun et al. (2021) *China*	Same sample 12,889 (80%) 20 (NA) NA	NA	T1 = 12/2019–01/2020 T2 = 05/2020	7.4%	CNSPFS Battery BDI-II	Anaerobic, aerobic, explosive and muscular fitness were independently and inversely associated with depression for the overall population
Elmer et al. (2020) *Switzerland*	Different samples 58 (38.9%) 212 (NA) NA NA	Engineering and Natural Science (2° and 3° academic year)	T1 = 04/2019 T2 = 09/2019 T3 = 04/2020	NA	Cohort network items Personal network items COVID-19 items CESDS GAD-7 PSS-10 UCLA-LS BFI	Co-studying network has become sparser during the pandemic. Students’ level of stress, anxiety, loneliness and depressive symptoms were higher than before the pandemic. Female reported worse mental health trajectories
Fruehwirth et al. (2021) *USA*	Same sample 419 (NA) 18.9 (NA) Non-Hispanic White 61.9%	NA	T1 = 10/2019–02/2020 T2 = 06/2020–07/2020	58%	PHQ-8 GAD-7 Items about COVID-19 stressors BRS BRCS MSPSS	Increase in anxiety and depression prevalence during the pandemic compared to baseline assessment. White, female, Sexual and Gender Minorities (SGMs) were at highest risk of increases in anxiety symptoms. Non-Hispanic black, female and SGM students were at highest risk of increases in depression symptoms. General difficulties associated with distanced learning and social isolation contributed to the increases in both depression and anxiety symptoms
Hamza et al. (2021) *Canada*	Same sample 733 (74%) 18.5 (0.7) Caucasian 21%	NA	T1 = 05/2019 T2 = 05/2020	23.9%	ICSRLE MSPSS PSS-10 DERS-SF PANAS-X ISAS CESDS-R GAD-7 MSI-BPD AUDIT PCL PBS GSCA	Students with pre-existing mental health concerns showed improving or similar mental health during the pandemic (compared with one year before). In contrast, students without pre-existing mental health concerns were more likely to show declining mental health, which coincided with increased social isolation
Horita et al. (2021) *Japan*	Different sample 400 (56.6%) 766 (45.4%) NA NA	NA	T1 = 15/04/2019–31/05/2019 T2 = 20/04/2020–31/05/2020	Not applicable	K10 CCAPS-Japanese	The number of “high-risk” students and the depression level were lower among the 2020 first-year students compared to previous year’s students
Huckins et al. (2020) *USA*	Same sample 217 (67.8%) NA NA	NA	SS = 08/2017 SE = 03/2020	17.9%	Mobile sensing (sedentary time, sleep, location, phone usage, COVID-19 news coverage) PHQ-4	During winter 2020 term, students were more sedentary and reported increased anxiety and depression symptoms than in previous academic terms and subsequent academic breaks
Li et al. (2020a) *China*	Same sample 555 (76.8%) 19.6 (3.4) NA	NA	T1 = 12/20/2019 T2 = 02/2020	None	PANAS PHQ-4	Increase in negative affect and symptoms of anxiety and depression after 2 weeks of confinement
Li et al. (2020b) *China*	Same sample 173 (75.8%) 19.8 (0.9) NA	Social Work, International Economics, Economics and Trade, Marketing and Accounting (2° and 3° academic year)	T1 = 20/11/2019–28/11/2019 T2 = 28/02/2020–10/03/2020 T3 = 29/05/2020–10/06/2020	12.3%	LMS C-DASS-2	Stress, anxiety, and depression all showed V-shaped growth trajectories in which these variables decreased during lockdown, before increasing in the post-lockdown period
Meda et al. (2021) *Italy*	Different samples 161 (NA) 197 (NA) NA NA	Medicine and Surgery, Psychology, Biology, Pharmacy, Economics, Engineering, Social and Political Sciences NA	Sample 1 T1 = 10/2019 T2 = 03/04/2020–23/04/2020 Sample 2 T1 = 11/11/2019–19/12/2020 T2 = 11/05/2020–21/06/2020	NA NA	BDI-2 BAI OCI-R EHQ EDI-3	Students reported worse depressive symptoms during lockdown than 6 months before isolation, with students without previous diagnosis of psychopathology. being affected the most
Rettew et al. (2021) *USA*	Same sample 484 (76%) 18.1 (0.3) White 90%	NA	SS = 01/2020 SE = 05/2020	28.1%	BFI EMA (5 well-being items, 2 mood items)	Mood and well-being indices declined during the COVID-19 pandemic, while stress decreased. Differential impacts of the COVID-19 outbreak for students with low versus high levels of particular personality traits
Saraswathi et al. (2020) *India*	Same sample 217 (64%) 20.0 (1.6) NA	Medicine and Surgery NA	T1 = 12/2019 T2 = 06/2020	7%	DASS-21 PSQI	Increase in both prevalence and levels of anxiety and stress
Van Zyl et al. (2021) *Netherlands*	Same sample 141 (31.9%) NA NA	NA	SS = 01/2020 SE = 04/2020	None	SDRS MHC-SF	Mental health was reported to be moderate and stable throughout the study
Wilson et al. (2021) *USA*	Different samples 1019^b^ (64.6%)^a^ 20.9 (1.5) White 80.3%	NA	SS = 01/2015 SE = 04/2020	NA	GPAQ PSS-4 CESDS-7	Decrease in physical activity during the COVID-19 pandemic. Perceived stress increased, and depressive symptoms also increased among female in the same period
Zhang et al. (2020) *USA*	Same sample 49 (NA) NA NA	NA	T1 = 01/2020–02/2020 T2 = 03/2020–05/2020	None	PHQ-9 GAD-7 Google and YouTube searches analysis through 5 features	Increase in depression and anxiety. Online behavior features were significantly correlated with deteriorations in PHQ-9 and GAD-7 scores

*NA* non-available; *MA* mean age; *SS* study start; *SE* study end; *AUDIT* Alcohol Use Disorder Identification Test; *BAI* Beck Anxiety Inventory; *BDI* Beck Depression Inventory; *BFI* Big Five Inventory; *BPM/18–59* Brief Problem Monitor; *BRCS* Brief Resilient Coping Scale; *BRS* Brief Resilience Scale; *CCAPS* Counselling Center Assessment of Psychological Symptoms; *CCSM* Cross-Cutting Symptoms Measure; *CESDS* Center for Epidemiological Study of Depression Scale; *CNSPFS Battery* Chinese National Student Physical Fitness Standard; *DASS* Depression Anxiety Stress Scale; *DERS-SF* Difficulties in Emotion Regulation Scale- Short Form; *EDI-3* Eating Disorder Inventory-3; *EHQ* Eating Habits Questionnaire; *EMA* Ecological Momentary Assessment; *GAD* Generalized Anxiety Disorder; *GPAQ* Global Physical Activity Questionnaire; *GSCA* Grit Scale for Children and Adults; *ICSRLE* Inventory of College Students’ Recent Life Experiences; *ISAS* Inventory of Statements about Self-Injury; *K10* Kessler Psychological Distress Scale; *LMS* Langer Mindfulness Scale; *MHC-SF* Mental Health Continuum-Short Form; *MSI-BPD* McLean Screening Instruments for Borderline Personality Disorder; *MSPSS* Multidimensional Scale of Perceived Social Support; *OCI-R* Obsessive–Compulsive Inventory Revised; *PANAS* Positive And Negative Affect Schedule; *PBI* Parental Bonding Instrument; *PBS* Perceived Burdensomeness Scale; *PCL* Posttraumatic Stress Disorder Symptoms Checklist; *PHQ* Patient Health Questionnaire; *PSQI* Pittsburgh Sleep Quality Index; *PSS* Perceived Stress Scale; *RQ* Relationship Questionnaire; *SCL-90-R* Symptom Checklist 90 Revised; *SDRS* Study Demands and Resources Scale; *STAI* State Trait Anxiety Inventory; *UCLA-LS* UCLA Loneliness Scale

^**a**^Sample characteristics refer to the totality of participants

^b^Cumulative sample

The studies varied in their sample sizes (from 49 up to 12,889 college students) and all together included a total of 20,108 subjects, most commonly female. The average sample age was 19.6 years (calculated only for studies reporting the age of students). Six studies reported the course studied by the college students: the participants studied Psychology (Charles et al., 2021; Meda et al., 2021), Medicine and Surgery (Meda et al., 2021; Saraswathi et al., 2020); Agriculture (Li et al., 2020a), Engineering (Elmer et al., 2020; Meda et al., 2021), Economics (Li et al., 2020b; Meda et al., 2021), Natural Sciences (Elmer et al., 2020), Social Work (Li et al., 2020b), Marketing (Li et al., 2020b), Accounting (Li et al., 2020b), Biology (Meda et al., 2021), Pharmacy (Meda et al., 2021), and Social and Political Sciences (Meda et al., 2021). Few of the studies reported students’ academic year at the time of assessment, i.e. second or third year (Elmer et al., 2020; Li et al., 2020b). The studies included covered a period from January 2015 as the earliest baseline (Wilson et al., 2021) to December 2020 as the latest follow-up (Meda et al., 2021). Drop-out rates at follow-up among the included studies go from a minimum of 7.0% (Saraswathi et al., 2020) to a maximum of 58% (Fruehwirth et al., 2021). Ten out of 17 studies (58.8%) focused only on mental health related variables; the remaining seven studies focused on both mental health and lifestyle (e.g. physical activity, internet and smartphone use, etc.).

### Changes in Mental Health and Well-Being During the COVID-19 Pandemic

The majority of the included studies reported a worsening of mental health and a decline in well-being in college students during the COVID-19 pandemic. Most of the studies found an increase in anxiety symptoms (Bussone et al., 2020; Charles et al., 2021; Elmer et al., 2020; Fruehwirth et al., 2021; Huckins et al., 2020; Li et al., 2020a; Saraswathi et al., 2020; Zhang et al., 2020), depression and mood disorders (Bussone et al., 2020; Charles et al., 2021; Copeland et al., 2021; Elmer et al., 2020; Fruehwirth et al., 2021; Huckins et al., 2020; Li et al., 2020a, 2020b; Meda et al., 2021; Rettew et al., 2021; Zhang et al., 2020), and personality disorders (Charles et al., 2021). The studies also reported, after the onset of COVID-19, an increase in distress (Bussone et al., 2020; Charles et al., 2021; Elmer et al., 2020; Horita et al., 2021; Li et al., 2020b; Saraswathi et al., 2020; Wilson et al., 2021), loneliness (Elmer et al., 2020), alcohol use (Charles et al., 2021), problems in externalization and attention (Copeland et al., 2021). It is also to be noted that, in some of the included studies, there was a decrease in psychological symptoms (Horita et al., 2021; Li et al., 2020b; Rettew et al., 2021) or a finding that psychological symptoms remained stable (Van Zyl et al., 2021) during the COVID-19 pandemic.

### Differences in Mental Health Among Subgroups During the COVID-19 Pandemic

Some of the included studies reported that mental health and well-being trajectories varied among social groups. Although both black and white students reported elevated symptoms during the COVID-19 pandemic (Charles et al., 2021; Fruehwirth et al., 2021), white students reported higher levels of anxiety (Charles et al., 2021; Fruehwirth et al., 2021), anger, sleep problems, perceived distress, and alcohol abuse (Fruehwirth et al., 2021), whereas black students reported higher levels of depression (Fruehwirth et al., 2021), mania, and psychosis than white students (Charles et al., 2021). Interestingly, female students reported higher levels of anxiety (Elmer et al., 2020; Fruehwirth et al., 2021), depression (Elmer et al., 2020; Fruehwirth et al., 2021; Wilson et al., 2021), stress and loneliness (Elmer et al., 2020). Sexual and gender minorities (SGMs) also reported higher levels of anxiety and depression during the COVID-19 pandemic (Fruehwirth et al., 2021).

### Risk and Protective Factors for Mental Health Issues During the COVID-19 Pandemic

Some studies identified the following possible risk factors of college student mental health during the COVID-19 pandemic: secure attachment and high levels of parental bonding (Bussone et al., 2020); high extroversion, openness, and agreeableness (Rettew et al., 2021). On the contrary, two studies (Hamza et al., 2021; Meda et al., 2021) reported that subjects without a history of psychological problems showed poorer mental health outcomes during the pandemic than students with pre-existing mental health problems. One study reported physical activity as a possible protective factor of college student mental health during the COVID-19 pandemic (Dun et al., 2021).

### Changes in Lifestyle During the COVID-19 Pandemic

The reported changes in college student lifestyle include: increase in sedentary behavior (Huckins et al., 2020); decrease in physical activity (Wilson et al., 2021); more time studying alone (Elmer et al., 2020); and increase in Internet use and changes in searched content on Google and YouTube (Zhang et al., 2020).

## Discussion

Understanding changes in mental health and lifestyle among college students during the COVID-19 pandemic requires longitudinal studies. This review of results obtained from longitudinal studies highlighted evidence of an increase in psychological symptoms (mostly anxiety, depression, distress, and loneliness) during the pandemic, when compared to data before the outbreak. Lifestyles also changed during the pandemic, with students engaged in more sedentary behavior, less physical activity, more and longer Internet use, and more time studying alone, with possible detrimental implications for their mental health.

This review showed an association between specific groups and mental health: female students and SGMs, in fact, showed poorer mental health outcomes (Elmer et al., 2020; Fruehwirth et al., 2021; Wilson et al., 2021). A possible explanation for this result is that women appear to rely on social support more than men (Tamres et al., 2002), while SGMs rely on their communities of equals and allies to cope with the difficulties of discrimination and prejudice (de Lira & de Morais, 2018). During the pandemic, because of measures implemented to curb the spread of the virus, social interactions outside individual households were reduced to a minimum in many countries around the world, depriving the members of these groups of one of their main coping strategies. The COVID-19 pandemic also seems to have had an impact on both white and black students’ mental health, although with different symptoms (Charles et al., 2021). White students reported more anger, anxiety, sleep problems, perceived stress and alcohol use than African American students, who reported higher mania and psychosis symptoms than their white peers. This result replicated the findings of many studies, which had observed higher rates of psychotic disorders among people of African heritage (Strakowski et al., 1996, 2003; Bresnahan et al., 2007; Kirkbride et al., 2012). The reasons for these apparent differences are still unknown (Perlman et al., 2016) and may reflect both clinician bias (Neighbors et al., 2003; Trierweiler et al., 2006) and socioeconomic variables, which are associated with both ethnicity and risk of psychosis (Fearon et al., 2006; Sharpley et al., 2001).

Not all of the included studies reported an increase in psychological problems. However, in one case, the authors suggested that one of the reasons for this outcome could be the reduction in sample size during the follow-up assessment (Horita et al., 2021). Yet in some studies, symptoms decreased or remained stable (Hamza et al., 2021; Horita et al., 2021; Li et al., 2020b; Rettew et al., 2021; Van Zyl et al., 2021). Contrary to previous research which found that a pre-existing history of mental disorders was associated with a worsening of symptoms during the pandemic (Brunoni et al., 2021; Hao et al., 2020), some authors reported that students without previous mental health issues showed worse psychological outcomes than students with pre-existing mental health problems (Hamza et al., 2021; Meda et al., 2021). Meda et al. (2021) reported this result exclusively for depressive symptoms. The authors give two possible explanations for this result: regression toward the mean and differences in sociodemographic characteristics of the sample between baseline and follow-up. Given the longitudinal nature of the data and the absence of a control group (Rocconi & Ethington, 2009) and any reference to a change in relevant sociodemographic variables in their sample between baseline and follow-up, the result of Hamza et al. (2021) could also reflect the regression toward the mean. In fact, students with a pre-existing history of mental problems are more likely to score the highest during pre-test at measures assessing mental health issues. These individuals are likely to have achieved this position by having a high true value but also a very positive measurement error, which could push the result to the extreme (Rocconi & Ethington, 2009). If these individuals are re-assessed, it is highly unlikely that they will have such large positive error in measurement the second time, resulting in the second measurement being lower than the first. This will occur because within the normal distribution of errors of measurement, extremely large (or small) errors are in the tails of the distribution and less likely to occur than those at the center of the distribution (Rocconi & Ethington, 2009).

Personality traits, attachment style, parental bonding and physical activity are other factors that might explain the psychological adjustment of students during the pandemic. Extrovert, open, agreeable students, with secure attachment and high parental bonding, reported worse mental health outcomes during the pandemic. Extrovert subjects cope through social support and have larger and more diverse social support networks than introverts (Swickert et al., 2002), which may explain why they were more severely affected by lockdown/isolation. Moreover, the restrictions may not have been sufficiently stressful to trigger the stress response system of subjects with insecure attachment and low parental care (Bussone et al., 2020).

The results of this review, in agreement with previous research (Baker et al., 2010; Cangin et al., 2018; Kerling et al., 2015; Marques et al., 2020), revealed physical activity as a possible protective factor of college student mental health during the COVID-19 pandemic (Dun et al., 2021). The relationship between physical activity and well-being may have both a physiological and a psychological explanation; indeed, physical exercise seems to have a positive impact on the neurotransmitter system, regulating primary monoamines like dopamine, noradrenaline and serotonin (Dishman, 1997). It also seems to have a positive relationship with mental resilience (Childs & De Wit, 2014), self-efficacy and self-esteem (Blumenthal et al., 2007).

The results of this review partially replicated the findings of the meta-analysis by Deng et al. (2021). According to these authors, the pooled prevalence of depressive symptoms, anxiety symptoms, and sleep disturbances during the pandemic was 34%, 32% and 33%, respectively, with differences among subgroups (such as geographical regions, diagnostic criteria, education level, year of study, financial situation, living arrangements and gender). Deng et al. (2021) included mostly studies with a cross-sectional design in their review (in fact, only four out of 89 of their included studies have a longitudinal design), whereas this review only included studies with a longitudinal design, with baseline assessments before the outbreak of the COVID-19 pandemic. Given the longitudinal nature of these findings, this review replicates and even corroborates their results, except for sleep disturbances. One of the main findings of this review, in fact, is that students reported more symptoms during the pandemic than before. In addition, as reported in the study by Deng et al. (2021), this review found higher rates of psychological symptoms among female students than male students.

### Limitations of the Literature

The limitations of this review are important to consider. The quality of the studies included was quite low, and the samples were composed predominantly of female students from a single university. Moreover, in four studies, data on drop-out rates at follow-up were not available (Charles et al., 2021; Elmer et al., 2020; Meda et al., 2021; Wilson et al., 2021). For this reason, conclusions about the impact of the COVID-19 pandemic on student mental health should be interpreted with caution. Furthermore, the included studies did not report whether the students who dropped out of the study also dropped out of college. One of the main results of this review, in fact, is that students might have felt more stressed during the pandemic and stress can be a risk factor for dropping out of college. Another limitation is that only three out of 17 included studies reported control of confounding factors (Dun et al., 2021; Saraswathi et al., 2020; Zhang et al., 2020), and this may have reduced the comparability between subjects in different outcome groups. Finally, few studies (Charles et al., 2021; Copeland et al., 2021; Elmer et al., 2020; Fruehwirth et al., 2021; Huckins et al., 2020) included questions on COVID-19 infection (self, someone close). In light of this, it is impossible to separate the impact on student mental health of the restrictions and isolation imposed as a result of the pandemic from the possible traumatic effect of the direct experience of illness or the death of close relatives or friends.

### Limitations of the Review

Limitations of this review also are important to consider. Representativeness and sample size limitations reduce the generalizability of the results. Restricting the literature search to peer-reviewed studies published in English may have reduced the cultural diversity of the included studies. Even though Nussbaumer-Streit et al. (2020) found that the exclusion of non-English publications tends not to effect overall review conclusions, this remains a concern.

### Implications for Future Research

This review has highlighted the impact of the COVID-19 pandemic on the mental health and well-being of college students. The findings suggest that there was an increase in anxiety, depression, distress and loneliness during the pandemic. However, it is evident that further research is required to explore more fully the real impact of the COVID-19 pandemic on college students’ well-being and mental health. For instance, further studies should be conducted to investigate the impact of the pandemic also on college drop-out rates.

There are other factors that can correlate with student mental health, such as socio-economic status, living conditions, and physical health. These factors should be controlled in further studies in order to reduce the risk of bias in the interpretation of the results. The findings from this review provide early information toward the development of support interventions for college students to promote their well-being and mental health. In particular, these interventions should target certain student subgroups that are higher risk, such as female students and SGMs. The decline in mental health and well-being caused by the COVID-19 pandemic highlights the need to encourage help seeking among college students, possibly by planning public events promoting mental health symptom recognition and acceptance and destigmatizing mental illness among peers. Research also suggests the need to organize activities to improve stress management and lifestyle, promote physical exercise, and reduce addiction-related behavior, such as substance abuse and use of the internet/mobile devices.

## Conclusion

A large number of studies report on the impact of COVID-19 on the mental health of college students, but the scientific literature does not always include comparisons to pre-pandemic indicators. This review provides an overview of the changes in college student mental health, psychological well-being and lifestyles, with a focus on longitudinal studies. The findings highlight a worsening of mental health and a decline in well-being among college students during the pandemic. The results also show that mental health and well-being trajectories varied among social groups. In particular, female students and SGMs reported higher levels of anxiety and depression. Further research is needed to examine thoroughly the impact of the pandemic on vulnerable subgroups of college students.

## Supplementary Information

Below is the link to the electronic supplementary material.

Supplementary file1 (DOCX 16 KB)

## Declarations

### Preregistration

The protocol was registered with the Open Science Foundation (OSF) database. The protocol, which includes the research questions, detailed methods and planned analyses for the review, can be accessed at the following URL: https://osf.io/m6hyg/, 10.17605/OSF.IO/M6HYG (Date of registration: 31 May 2021).
