# Supplementary material for: Changes in College Students Mental Health and Lifestyle During the COVID-19 Pandemic: A Systematic Review of Longitudinal Studies
Source: Adolesc Res Rev. 2022 Aug 3;7(4):537–50. doi: 10.1007/s40894-022-00192-7 (PMC9362152; doi:10.1007/s40894-022-00192-7)
Supplement: Supplementary file 1 — Supplementary file1 (DOCX 16 KB) [file 40894_2022_192_MOESM1_ESM.docx]

**Table 1s**

| *Bibliographic database search strategies* |
| --- |
| Keywords |
| #1 “university student*” OR “college student*” OR “undergraduate student*” |
| #2 COVID-19 OR coronavirus OR SARS-CoV-2 |
| #3 longitudinal OR before OR after OR effect* OR impact |
| #4 well-being OR “mental health” |
| #1 AND #2 AND #3 AND #4 |
| PubMed |
| #1 [Title/Abstract] |
| #2 [Title/Abstract] |
| #3 [Title/Abstract] |
| #4 [Title/Abstract] |
| #1 AND #2 AND #3 AND #4 |
| EBSCO |
| #1 [Abstract] |
| #2 [Abstract] |
| #3 [Abstract] |
| #4 [Abstract] |
| #1 AND #2 AND #3 AND #4 |
| SCOPUS |
| #1 [Title/Abstract/Keywords] |
| #2 [Title/Abstract/Keywords] |
| #3 [Title/Abstract/Keywords] |
| #4 [Title/Abstract/Keywords] |
| #1 AND #2 AND #3 AND #4 |
| Web of Science |
| #1 [Title] |
| #2 [Title] |
| #3 [Title] |
| #4 [Title] |
| #1 AND #2 AND #3 AND #4 |
